# Experience With Pediatric Medical Device Development

**DOI:** 10.3389/fped.2020.00079

**Published:** 2020-04-07

**Authors:** H. David Humes, Angela J. Westover

**Affiliations:** Division of Nephrology, Department of Internal Medicine, University of Michigan Medical School, Ann Arbor, MI, United States

**Keywords:** child, medical device, humanitarian use device, orphan diseases, renal replacement therapy, immunomodulation

## Pediatric Medical Devices: Underserved and Largely Ignored

Few FDA approved medical devices are specifically designed for children's needs. FDA approval for clinical indications of medical devices specify procedures and not patient ages. Accordingly, the majority of both devices and drugs in pediatric patients are used for off-label indications. Data suggests that 60–75% of medical devices or drugs in pediatric patients are used for off label indications (1). This approach has drawbacks including safety and performance concerns with lack of proper education and instructions for the use of an adult device for a pediatric patient.

Barriers to pediatric medical device development arise from the small numbers of pediatric patients, numbered in the thousands vs. hundreds of thousands in the adult market. The number of cancer patients in pediatrics is ~2,000 vs. 600,000 adult patients; the number of defibrillators use in pediatrics is 1,600 vs. 200,000 in adult cardiology (1). Due to the low volume of patients, clinical trials in children have much slower enrollment than adult trials. Parental consent also complicates the enrollment of children in clinical research protocols. Liability concerns, although not discussed openly, may be another detriment for pediatric drug and device innovation.

To encourage pediatric device development, Congress and FDA established the Pediatric Medical Device Safety and Improvement Act of 2007 (PL-110-85). This allowed the FDA to designate a Humanitarian Use Device (HUD) designation for disorders with 4,000 patients annually and allowed a Humanitarian Device Exemption (HDE) marketing approval by the FDA for a device. This approval is based upon “safety and probable benefit” rather than the FDA standard Premarket Approval (PMA) process based upon randomized control trials demonstrating statistically significant “safety and effectiveness.” This approach was due to the low number of patients in many pediatric diseases to perform randomized control trials in a reasonable time frame. The elimination of the profit restriction on devices approved under an HDE also promoted financial incentives for pediatric device development (2). On December 2016 the twenty-first Century Cures Act (PL-114-255) changed the population estimate required to qualify for HUD designation from “fewer than 4,000” to “not more than 8,000” to further incentivize pediatric device development.

Even prior to these Congressional mandated incentives for pediatric devices, the passage of the Orphan Drug Act in 1983 (PL-97-414) also encouraged the development and approval of drugs for rare diseases. This Act established the Orphan Products Clinical Trials Grant Program in the FDA's Office of Orphan Products Development (OOPD) to support developing drugs and devices to treat orphan diseases. An orphan disease is defined as a disorder affecting fewer than 200,000 patients in the United States. Since developing a new drug or device is costly with inherent risk, large pharmaceutical drug companies have had little interest due to small market size and difficulty in recruiting sufficient number of subjects to study safety and efficacy of a new compound or device. Accordingly, this Act and its subsequent amendments in 1984, 1985, 1988, and 2007, provided a number of incentives for companies to develop compounds to treat rare diseases, including tax credits for the costs of clinical research, 7-year period of exclusive marketing after an orphan drug is approved, and waiver of Prescription Drug User Fee Act (PDUFA) filing fees (over $1 million).

With these incentives interest in orphan disease indications have occurred over the last decade (3, 4). This interest is driven also by the facts that there are ~7,000 rare diseases affecting 30 million people in the United States and 400 million worldwide and the recognition that many of these rare diseases have no effective treatments. Accordingly, small biotechnology companies have been formed with funding from private equity to develop new approaches to the unmet medical needs of orphan diseases due to high potential returns on investment. In fact, drugs to treat orphan diseases have commanded high price tags due to the small number of patients and non-competition (5). A recent study has shown that companies with regulatory approved orphan drugs are more profitable and are more attractive investment opportunities than companies without orphan drugs (6). With this background, the development of a potentially transformative device to treat adult and pediatric ICU patients with acute kidney injury requiring continuous renal replacement therapy(CRRT) provides an illustration of how the regulatory environment and the congressional legislation described above resulted in a pivoted focus on pediatric rather than adult indications. This opinion is based upon a singular experience in the bumpy road to commercialization of an immunomodulatory device named the Selective Cytopheretic Device (SCD).

## A Serendipitous Discovery

Many scientific discoveries have occurred due to chance observations by scientists with detailed background knowledge and an honest curiosity to understand the unexpected results of planned experiments (7). In this regard, an unanticipated result in a clinical trial led to a platform discovery to immunomodulate the detrimental effects of the activated innate immunologic system in both acute and chronic organ failures. This resulted in the development of a Selective Cytopheretic Device.

**Figure 1:**
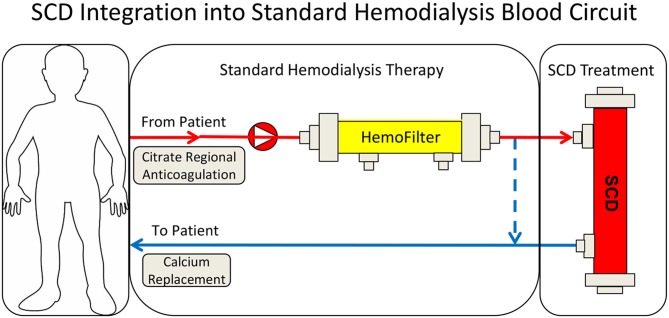
Schematic representation of the circuit used for selective cytopheretic device therapy.

The SCD originated from the clinical evaluation of a tissue engineered Renal Assist Device (RAD) (8) containing adult human renal epithelial cells as a component of a bioartificial kidney to provide more complete renal replacement therapy (RRT). The use of the metabolic activity of renal tubule cells was evaluated to assess whether this addition could improve the poor outcomes of ICU patients with severe acute renal failure requiring RRT. After safety and efficacy signals in Phase I/II and Phase II clinical trials, a change in clinical protocol was made in the RAD Phase IIb clinical study. Subsets of patients were treated with a cell containing RAD or a sham (non-renal cell containing) RAD cartridge (9). The Phase IIb study was a randomized control, blinded multicenter study in ICU patients with Acute Renal Failure secondary to Acute Kidney Injury (AKI) undergoing continuous renal replacement therapy (CRRT). The clinical study was suspended after an interim analysis due to an unanticipated high survival rate of the sham device arm. In retrospective analysis of the sham control groups, the improved survival rate was demonstrated in the presence of regional citrate anticoagulation (RCA) when compared to systemic heparin anticoagulation (10). Subjects were divided into four groups: (1) RAD with citrate anticoagulation, (2) sham device with citrate anticoagulation, (3) RAD with heparin anticoagulation, and (4) sham device with heparin anticoagulation. The 28-day survival rate in the heparin sham patient group was 50 vs. 75% in the citrate sham group (*n* = 12 for each treatment arm), and the 90-day survival rate was 25% (heparin) vs. 67% (citrate). The baseline demographics for the two subsets were comparable, with similar sequential organ failure assessment (SOFA) scores (13.4 ± 1.1 vs. 12.2 ± 0.9), organ failure number (4.17 ± 0.46 vs. 3.93 ± 0.36) and incidence of sepsis (58 vs. 58%) for the citrate vs. heparin sham groups, respectively (10). This clinical result, although unexpected, was consistent with a potential clinical benefit of the fiber based sham device without cultured renal cells (RAD sham), when used with RCA, which later became known as SCD therapy (Figure 1).

**Figure 2:**
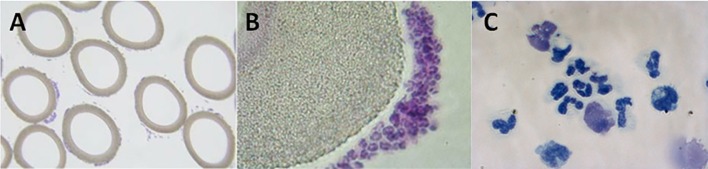
Micrographs of cross-sectional area of sham, acellular cartridges (as part of the regional citrate anticoagulation arm of the Renal Assist Device clinical trial now known as the selective cytopheretic device) **(A,B)**. Low-power micrograph showing adherent cells around each fiber (**A**, 4× objective). Higher-power micrograph showing clustering of bound leukocytes (**B**, 20× objective). High-power micrograph of a cytospin prepared from adherent cells washed from the outer membrane of the SCD after 24 h of therapy on the first pediatric patient (**C**, 63× objective). Patient treatment demonstrated adherent leukocytes with a predominance of neutrophils and monocytes on the outer surface of the membranes along the blood flow path which translated into patient benefit.

The therapeutic benefit afforded by this combination of a device and a compound (citrate) on a systemic clinical disorder can be better understood from the following: (1) Microscopy of the sham cartridges (future SCD) after patient treatment demonstrated adherent leukocytes on the outer surface of the membranes of the cartridge along the blood flow path (Figures 2A,B) (9). The attached leukocytes were dominated by neutrophils and monocytes (Figure 2C), which preferentially adhere, compared to other leukocytes such as lymphocytes (11). The ability of leukocytes to adhere to the outer walls of the hollow fiber membranes rather than the inner walls, which is the conventional blood flow path for renal dialysis/hemofiltration applications, was due to the shear forces of blood flow. The shear stress of blood along the outer wall of the membrane was near capillary force of <1 dyne/cm^2^ compared to the shear stress of 100 dyne/cm^2^ for blood flowing along the conventional luminal surface of the hollow fiber membranes. (2) RCA lowers the iCa in blood within the circuit to <0.4 mM, a level which inhibits the coagulation system, has an inhibitory effect on leukocyte and platelet activation (11, 12), and also affects the calcium-dependent selectin and integrin mediated interactions between leukocytes and the membrane (13, 14). Extravasation of neutrophils and monocytes from the systemic circulation into tissues is a highly regulated process. In a low shear force environment like that found in capillaries or created within the SCD, neutrophils and monocytes roll along surfaces and are slowed via selectin binding followed by integrin mediated firm adhesion prior to diapedesis (13).

Data from an *in vitro* blood study utilizing flow chambers to visualize leukocyte interactions with fiber materials suggested that leukocytes roll, then adhere to fibers, are retained for a significant time period (11) (referred to as sequestration) and are then released. Binding selectivity for more activated leukocytes in the SCD is increased in the low iCa environment where calcium dependent selectin rolling, integrin binding, and downstream conformational changes of attached cells are inhibited (15). Neutrophils (16, 17) and monocytes (18, 19) mobilize intracellular stores of CD11b, to the cell surface as they become (primed) activated. Measurement of CD11b, allows for real time measurement of systemic acute neutrophil (priming) and monocyte activation. Additionally, monocyte populations are heterogeneous in their expression of CD11b, with CD14^hi^CD16^−^ being the highest, and CD14^low^CD16^+^ being the lowest (20). It follows that the preferential sequestration of inflammatory CD14^hi^ monocytes is enhanced in the low iCa environment. The selectivity of binding of the highest activated leukocytes has been repeatedly observed in preclinical animal models where systemic CD11R3: the porcine analog of human CD11b (21), levels decrease through the treatment course (10, 11, 22, 23). This effect was measured directly in a clinical trial by comparing the CD11R3 relative fluorescence of the circulating cells in the peripheral blood to those directly associated with the SCD (24). These results when taken together (10, 11, 22–25), suggest a SCD mechanism of action with a simultaneous, combination effect to transiently sequester activated circulating neutrophils and monocytes, with enhanced selectivity for inflammatory leukocytes, which alters the overall activation of bound and processed leukocytes. Clinical efficacy in AKI with Multi-Organ Dysfunction (MOD) may be due to sequestration and immunomodulation of leukocytes in the SCD. This process appears to block the inflammatory sequence associated with accumulation and aggregation of leukocytes in the peritubular capillaries and reduce infiltration into interstitial spaces, that when unchecked promotes kidney injury following systemic inflammatory response syndrome (SIRS).

## Clinical Development of the SCD: Adult Trials

Preclinical large animal studies confirmed the efficacy of the SCD in a porcine model of septic shock with concomitant acute tubular necrosis (26). Product development continued with successful Phase I/II and Phase II clinical studies which demonstrated safety and strong signals for efficacy in ICU patients with AKI (11, 27). Accordingly, a phase III multi-center, randomized, controlled, pivotal study to assess the safety and efficacy of a SCD in patients with AKI (IDE G090189, Protocol SCD-003) (28)was initiated. The primary objective of this study was to determine whether CRRT+SCD therapy, compared to CRRT alone, results in a clinically relevant and statistically significant improvement in all-cause mortality through day 60. Secondary objectives included assessment of RRT dependency at day 60, mortality at day 28, number of ventilator free days at day 28, and mortality at day 60 of the subset of patients with severe sepsis. This study was a two-arm, randomized, open-label, controlled multi-center pivotal study that enrolled 134 patients at 21 US medical centers. ICU AKI patients of each participating hospital were randomized to treatment undergoing CRRT or CRRT+SCD. Each participating clinical site used their established RCA protocol for the CRRT+SCD circuits (Study Arm) and for the CRRT only (Control Arm). The recommended iCa (riCa) level (measured post SCD) in the CRRT and SCD circuit was specified to be between 0.25 and 0.4 mmol/L.

During the second quarter of the enrollment period, a national calcium shortage occurred in the US from FDA related quality manufacturing issues of the major US supplier. This shortage resulted in most centers unable to recruit to the study, since injectable calcium is required for RCA. Due to reliance of the SCD on a narrow intra-circuit iCa range for functional efficacy and the concern that patients randomized to SCD therapy were not getting effective therapy, the interim analysis was performed early-after enrollment of 134 patients. Enrollment was paused on May 24, 2013, to assess the clinical impact of the calcium shortage on study endpoints. The shortage of calcium infusion solutions resulted in a tendency to minimize citrate infusion rates. Accordingly, iCa levels within the blood circuit tended to be above the recommended (r)iCa of 0.25–0.40 mmol/L. Subsequently, the injectable calcium shortage resulted in 9 of the 21 open clinical sites being unable to enroll patients due to low hospital inventories of injectable calcium, contributing to the early termination of the study. Of the 134 patients in the analysis, 69 received CRRT alone and 65 received SCD therapy. No significant differences were noted between the control and treatment groups in baseline characteristics. No statistically significant difference was found between the treated and control patients with a 60-day mortality of 39% (27/69) and 36% (21/59), respectively, with six patients lost to follow up. The amount of time the patients in both the control and treatment group were maintained in the riCa range (0.25–0.40 mmol/L), as specified in the study protocol, was substantially lower than expected due to the injectable calcium shortage. Of the 134 patients enrolled at the time of the interim analysis, 19 SCD patients and 31 control patients were maintained at riCa for ≥90% of the therapy time. Furthermore, none of the significant adverse events (SAE) were considered device related per the principal investigator and the Data Safety Monitoring Board. Comparison of these subgroups of patients revealed 60-day mortality was 16% (3/19) in the SCD group compared to 41% (11/27) in the control group (*p* = 0.11). Dialysis dependency showed a borderline statistically significant difference between the SCD vs. control patients maintained for >90% of the treatment in the protocol's riCa target range with values of 0% (0/16) and 25% (4/16), respectively (*p* = 0.10). When the riCa SCD and control subgroups were compared for a composite index of 60-day mortality and dialysis dependency, the percentage in SCD subjects was 16 vs. 58% in the control subjects (*p* < 0.01). When the riCa subpopulation was considered, a statistically significant difference was detected in several parameters: log urine output substantially increased, and absolute leukocyte and neutrophil counts diminished in the SCD vs. control groups over time (28).

## Adult Clinical Trials Summary

The observation that, in those patients who had the riCa level >90% of the time of SCD treatment, mortality improved from 41 to 16%, is clinically compelling. In addition, the observation both that in SCD clinical trials no patient receiving appropriate SCD therapy was dialysis dependent at day 60 is also compelling. Previous large prospective clinical studies in AKI with MOD had >20% incidence of dialysis dependency of patients followed for 60 or more days (29, 30). The effect of SCD therapy to modulate excessive leukocyte activation most likely plays a critical role in the recovery of renal function after a substantive AKI event. The relationship of ongoing inflammation in the kidney after AKI and chronic progressive kidney disease and dialysis dependency has been demonstrated (31, 32). In this patient population, immunomodulation by SCD therapy appears to positively promote kidney healing as evidenced by the lack of dialysis dependency at day 60. Additionally, improvement in overall mortality may suggest improved immune balance that persists through the late SIRS process to ameliorate the compensatory anti-inflammatory response which follows the excessive systemic pro-inflammatory state in AKI and MOD (33). Furthermore, the significant decrease in absolute leukocyte and neutrophil counts, as well as the improvement in urine output over time corroborates the mechanistic and pilot studies previously published (11, 27, 34).

## Pivot to Pediatric Devices

With this compelling *post-hoc* analysis, the company, Cytopherx, which licensed this technology from the University of Michigan to commercialize this therapy, underwent a diligent attempt to obtain private equity to undertake a final Premarket Approval (PMA) clinical trial to use the composite index of 60-day mortality and dialysis dependency for FDA approval and rights to market this device in the United States. This effort proved to be difficult with venture capital and private equity firms hesitant to commit tens of millions of dollars to undertake a final multicenter randomized, control study which failed in the initial attempt. Despite the compelling *post-hoc* analysis, and the lessons learned regarding careful control of the circuit iCa in the recommended range of 0.25–0.4 mM, the perception of a previously failed trial (minimizing the *post-hoc* analysis) and the risk of capital was too high of a hurdle to obtain commitment to fund the clinical program to achieve FDA approval.

With the failure to obtain funding commitments but being convinced from the compelling preclinical and the safety and efficacy clinical data from adult trials, our group considered testing SCD therapy in the pediatric population for a number of reasons. Since the pediatric patient with AKI and MOD usually is not saddled with various chronic diseases which may cause mortality within 60 days of recovery from AKI and dialysis, this patient population would have less obfuscating co-morbidities. An efficacy signal would be apparent in lesser number of patients, thereby confirming the *post-hoc* analysis of the Phase III adult trial. In addition, the route to FDA approval would not require a large number of patients due to a Humanitarian Use Designation (HUD) since there are <8,000 pediatric patients with AKI and MOD requiring CRRT annually in the United States. Upon demonstrating safety and probable benefit in this HUD pediatric trial, a Humanitarian Device Exemption (HDE) approval by the FDA will allow marketing and commercial sale of the SCD in the United States. Upon HDE approval, funds derived from private equity or public markets to carry out the PMA adult clinical trial would be more readily obtained.

With this strategy, our group contacted the prospective pediatric (pp) CRRT consortium (35, 36) directed by Dr. Stuart Goldstein, who agreed that this direction was feasible. Accordingly, our group with the collaboration of Dr. Goldstein, submitted an FDA Office of Orphan Products Development (OOPD) grant to carry out this clinical study. Funding was received in 2014 and the trial was initiated in 2015.

Accordingly, similar to the adult AKI clinical trial, a multicenter US study of the SCD in critically ill children (>15 kg, age up to 22 years) with AKI and MOD receiving CRRT as part of standard of care was initiated and is on-going under the FDA approved IDE#G150179 (clinicaltrials.gov NCT02820350). Mortality rates in pediatric patients with AKI and MOD requiring CRRT has historically approached 50% (35–37). In this clinical trial, pediatric patients have received SCD therapy for up to 7 days or when CRRT is discontinued, whichever comes first. Interim analysis of the 14 patients treated with the SCD revealed compelling safety and efficacy data similar to the *post-hoc* analysis of the Phase III adult SCD study of patients treated per protocol with the recommended iCa levels below 0.4 mM ninety percent of treatment time. The 14 treated patients had an age range between 5 and 20 years, had multiorgan failure between 2 and 5 organs, averaging 2.92 organ failures as a group. Eight of fourteen treated patients also presented with severe sepsis or septic shock. All patients received RCA per protocol with the recommended iCa levels below 0.4 mM for 90% of measured values during treatment. When compared to the historical control standard of care CRRT treatment of pediatric patients with AKI/MOD, SCD therapy reduced both 60-day mortality and ICU length of stay. No patient was dialysis dependent at 60 days. These results, therefore, support a plan to submit an HUD/HDE application to the FDA. These data also strongly support the *post-hoc* analysis of the adult study. A final IDE adult study using a composite outcome measure of 60-day mortality or 60-day dialysis independence has been approved by the FDA and successful fundraising is anticipated to move this therapy back to the large adult market which comprises of 160,000 patients in the U.S. on an annual basis.

## Summary

This case study demonstrates that creative strategic planning, recognition of FDA pathways and support for pediatric devices can coalesce to promote the development of a life saving device reaching the bedside to save lives and save hospital costs with decreasing length of stays. The product development of pediatric therapies may provide a unique opportunity to more clearly demonstrate the potential effectiveness of a therapy with a smaller population due to the lack of complications and comorbidities as is often seen in adult disease. The development of a pediatric therapy not only is ethically sound, but can also lead to easier and faster transition into the adult market negating the initial hesitancy from a perceived limited market. This case study provides a perspective of the clinical development of a pediatric device as an important step in the commercialization of an innovative therapy.

## Author Contributions

HH developed the main conceptual ideas of this manuscript. HH and AW contributed to the design, implementation of the supporting research, and writing the manuscript.

### Conflict of Interest

HH retains equity interest in Innovative BioTherapies and SeaStar Medical (formerly Cytopherix), the company licensed by the University of Michigan to commercialize the Selective Cytopheretic device technology described in this review. AW has equity interest in SeaStar Medical.
